# A closer look at the epidemiology of schizophrenia and common mental disorders in Brazil

**DOI:** 10.1590/1980-57642020dn14-030009

**Published:** 2020

**Authors:** Randhall Bruce Carteri, Jean Pierre Oses, Taiane de Azevedo Cardoso, Fernanda Pedrotti Moreira, Karen Jansen, Ricardo Azevedo da Silva

**Affiliations:** 1 Department of Health and Behavior, Universidade Católica de Pelotas - Pelotas, RS, Brazil.; 2 Centro Universitário Metodista IPA - Porto Alegre, RS, Brazil.; 3 Institute of Biosciences, Universidade Federal do Mato Grosso do Sul - Campo Grande, MS, Brazil.

**Keywords:** mental health, anxiety, affective disorders, bipolar disorder, depression, saúde mental, ansiedade, transtornos do humor, transtorno bipolar, depressão

## Abstract

Schizophrenia and common mental disorders are noteworthy social and economic concern worldwide. Epidemiologic studies on the impact of specific mental disorders in emerging countries are scarce.

**Objectives:**

We aimed to characterize the demographic, social, and economic burden of schizophrenia and common mental disorders patients in the health system in Brazil.

**Methods:**

Data on these conditions in Brazil between 2008 and 2019 were collected through the website of the Departamento de Informática do Sistema Único de Saúde (Information Technology Department of the Unified Health System - DATASUS) maintained by the Brazilian Ministry of Health. Mean annual hospital admissions were 154,009.67, and cumulative incidence of 77.44 admissions per 100,000 inhabitants.

**Results:**

Average annual hospital expenses were US$ 67,216,056.04, with an average admission cost of US$ 432.58. The most affected age groups were older adults albeit younger individuals showed a trend towards increase of occurrences in recent years. There were a higher number of admissions in men compared to women.

**Conclusions:**

We consider the results obtained important to assist in evaluating and guiding public policies regarding the prevention and treatment in health systems.

## RESUMO.

A esquizofrenia e os transtornos mentais comuns são uma preocupação social e econômica notável em todo o mundo. Estudos epidemiológicos sobre o impacto de transtornos mentais específicos em países emergentes são escassos.

**Objetivos:**

Nosso objetivo foi caracterizar a carga demográfica, social e econômica de pacientes com esquizofrenia e transtornos mentais comuns no sistema de saúde no Brasil.

**Métodos:**

Os dados sobre essas condições no Brasil entre 2008 e 2019 foram coletados por meio do site do Departamento de Informática do Sistema Único de Saúde (DATASUS), mantido pelo Ministério da Saúde do Brasil. As internações hospitalares anuais médias foram de 154.009,67, e a incidência acumulada de 77,44 internações por 100.000 habitantes.

**Resultados:**

As despesas hospitalares médias anuais foram de US$ 67.216.056,04, com um custo médio de internação de US$ 432,58. As faixas etárias mais afetadas foram os adultos mais velhos, embora os mais jovens tenham demonstrado tendência a aumento de ocorrências nos últimos anos. Houve um número maior de admissões entre os homens em comparação às mulheres.

**Conclusões:**

Consideramos importantes os resultados obtidos para auxiliar na avaliação e orientação de políticas públicas de prevenção e tratamento nos sistemas de saúde.

## INTRODUCTION

Schizophrenia and common mental disorders are characterized by cognitive, emotional and behavioral disturbances which could culminate in impaired mental functioning, while potentially affecting every stage of life, from childhood and adolescence through adulthood.^1^ According to the Diagnostic and Statistical Manual for Mental Disorders, 5^th^ edition (DSM-V), schizophrenia is characterized by delusions, hallucinations, disorganized speech and behavior, and other symptoms that cause social or occupational dysfunction. For a diagnosis, symptoms must have been present for six months and include at least one month of active symptoms.^1^ On the other hand, mood disorders could account for depression and bipolar disorder; an episode of depression is characterized by the presence of distinct episodes lasting at least two weeks (although most episodes last considerably longer) involving clear changes in affection, cognition and neurovegetative functions, and interepisode remissions, albeit bipolar disorder is characterized by presence of a prominent and persistent period of abnormally high, expansive or irritable mood and abnormally increased activity or energy predominating in the clinical picture attributable to another medical condition. The manic episode may have been preceded or followed by hypomanic or major depressive episodes. In addition, a major issue during the development of the DSM-V and a conflicting topic in literature is whether schizophrenia or psychotic mood disorders are different disorders, or whether they constitute a “psychosis continuum".^2^ Therefore, since schizophrenia and different mood disorders can share several clinical similarities, it would be appropriated to consider these conditions together to explore their occurrence regarding hospitalizations.^3^

Further, common mental disorders often includes depression, generalized anxiety disorder, panic disorder, phobias, social anxiety disorder, obsessive-compulsive disorder and post-traumatic stress disorder, not necessarily including conditions related to drug abuse.^4^ ^,^ ^5^ Hence, considering the high comorbidity level between several mental disorders, psychiatric epidemiology commonly reports the prevalence of combined disorders, since data can improve transdiagnostic interventions and the effectiveness of mental health policies.^6^

According to the World Health Organization (WHO), the number of people with common mental disorders globally is increasing, particularly in lower-income countries, due to population growth and aging.^7^ Recently, WHO adopted a special initiative to advance in policies and interventions to ensure quality of care for patients with impaired mental health conditions.^8^ This epidemiologic approach provides appropriate information to introduce primary (avoiding the occurrence), secondary (rapid and proper treatment to reduce disease impact) and tertiary prevention (mitigating the disability and reducing the individual limitations caused by these conditions).^8^

Data obtained in Brazil indicates that these disorders account for 21.5% of all disability-adjusted life years in the country.^9^ In addition, São Paulo ranks among cities with highest prevalence of mental health conditions around the world.^10^ Aiming at improving mental health, Brazilian government increased investments in mental health services and health care. This led to integration of mental health into primary care and increasing the population access to mental health care with the Family Health Strategy Program.^11^ Since most of epidemiologic studies in Brazil were conducted in combined primary health centers^12^ or specific cities,^10^ ^,^ ^13^ a broader evaluation of available nationwide data would provide further insights to future directions for public health policies.

Therefore, given the importance of the theme, scarcity of data in the scientific literature, and growing need for specific epidemiologic vigilance policies for schizophrenia and common mental disorders in Brazil and worldwide, here we aimed to characterize the demographic, social, and economic burden of these specific disorders in the health system in Brazil, using data provided by the Departamento de Informática do Sistema Único de Saúde (Information Technology Department of the Unified Health System -DATASUS).

## METHODS

This is a population study, based on descriptive statistics to characterize schizophrenia and common mood disorders in Brazil from 2008 to 2019. Therefore, approval of the Ethics Committee in Research is considered dispensable since all data was obtained from public domain database, accessible online.

Analysis of the data available from January 2008 to December of 2019 was performed. All information used to analyze the profile of schizophrenia and common mood disorders in Brazil came from the database of the DATASUS (available online at http://www2.datasus.gov.br). This database is fed by the “hospital admission authorization (HAA)” by both public and private health institutions composing the Sistema Único de Saúde (Unified Health System - SUS) in Brazil, as all institutions send information on hospitalizations made through the HAA to municipal and state managers, which provide all data for consolidation in the database.

Based on the International Disease Classification, 10^th^ Revision (ICD-10), the terms “Affective and Mood Disorders” (F30-F39), “Schizophrenia” (F20-F29), “Stress-Related disorders” (F40-F48) and “Other mental health disorders” (F04-F09; F50-F69; F80-F99) were selected from a list of diagnoses. In addition, we chose to exclude mental health disorders related to drugs of abuse or alcohol consumption, dementia, mental retardation, Alzheimer or Parkinson diseases. These diagnostic terms were related to common mood disorders among the options available for the research and, as such, these terms were chosen on the basis of the WHO recommendations.^14^

Data provided by DATASUS were selected to obtain total admissions occurrences and related costs. The costs (hospital services and professional services) are direct costs indicated and approved by the hospital and imputed in the database (therefore not indicating indirect costs), and costs in dollars were calculated from the value obtained in Brazilian real divided by 4.5 (dollar value compared to Brazilian real). These data were further discriminated by region of occurrence, year, sex and age group. The incidence (number of new cases in the population per year) was calculated with information of total resident population and age distribution of the population for each year, obtained by the agency responsible for official collection of statistical information in Brazil, Instituto Brasileiro de Geografia e Estatística (IBGE - available online at https://www.ibge.gov.br/). Graphics were obtained with Prism 7.0 software. Since we cannot obtain data from specific patients, hospital incidence may account for a single patient with several admissions.

Finally, we provided r^2^ values for specific observed trends using linear regression considering a significance level of p<0.05 and forecasted data using modelers for time series with Statistical Package for the Social Sciences, version 25.0 for windows.

## RESULTS

**Figure 1.**
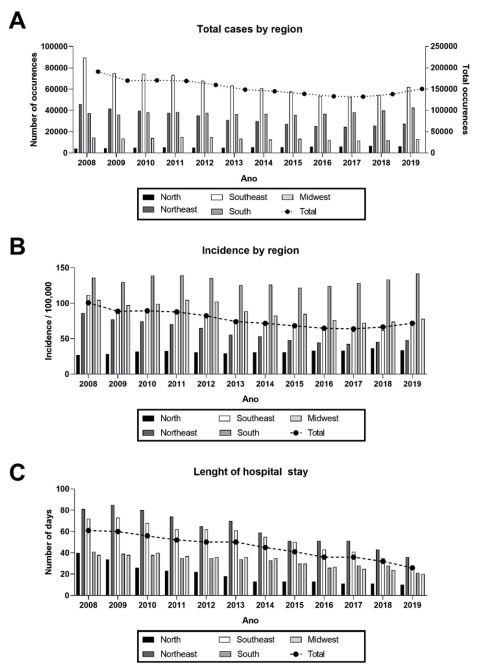
Total hospital admissions due schizophrenia and common mental disorders (A), incidence (B) and length of hospital stay (C) by region and year. Data for each region is plotted in the left Y axis, while total data (all regions combined) is plotted in the right Y axis.

There were a mean of 154,009.67 hospital admissions per year from 2008−2019 due to schizophrenia and common mental disorders in Brazil. The incidence of admissions was 77.44 per 100,000 inhabitants during the period, considering a population of 199,899,051.83 million inhabitants, calculated with mean estimates of Brazilian population from 2008−2019. Higher incidence is observed in the South (131.75) followed by Midwest and Southeast (88.67 and 78.06, respectively). Incidence was lower for Northeast (59.23) and North (31.43). Incidence for each region by year is shown in Figure 1A.

Analysis of the available DATASUS data from 2008−2019 showed that total hospital admissions were 1,848,116.00. In absolute numbers, the region with higher admission number was the Southeast (782,664.00) followed by South and Northeast (452,656.00 and 390,451.00, respectively). Average annual all-in costs of hospital expenses for patients suffering from schizophrenia and common mental disorders were around US$ 67,216,056.04 with an average cost per admission of US$ 432.58. On average, total days in hospital annually was 44.44 (Table 1). The length of hospital stay for each region by year is shown in Figure 1B, in which a trend towards decrease in for all regions is observed (r^2^=0.92).

**Table 1.** Admissions, costs and hospital days during 2008-2019 in Brazil.

	Admissions	Average costs	MHS cost	MHS
Brazil	154,009.67	US$67,216,056.04	US$432.58	44.44
North	5,331.25	US$974,312.45	US$188.91	17.71
Northeast	32,537.58	US$16,817,108.90	US$514.81	51.16
Southeast	65,222.00	US$34,809,763.73	US$525.79	56.13
South	37,721.33	US$10,757,449.44	US$285.14	27.78
Midwest	13,197.50	US$3,857,421.52	US$291.17	27.98

MHS: mean hospital stay (in days).

Data from 2008−2019 regarding the costs due to schizophrenia and common mental disorders showed a trend towards decrease from 2008 to 2017 (r^2^=0.79). However, the costs increased in 2018 and 2019 (r^2^=0.99). Therefore, we forecasted all costs for 2020 using prediction model with overall r^2^ values from 2008 to 2019 for each variable (Table 2). Overall costs increased compared to 2017, in all evaluated parameters, except for mean hospital stay cost, as showed in Table 2.

**Table 2.** Total costs due to schizophrenia and common mental disorder by year.

	Annual costs	Hospital services	Professional services	Mean of admission	MHS
2008	US$78,224,607.00	US$72,940,146.09	US$5,284,460.92	US$186.93	US$409.49
2009	US$81,760,994.40	US$75,553,214.29	US$6,207,780.09	US$202.00	US$481.24
2010	US$88,852,097.51	US$78,545,924.98	US$10,306,172.53	US$227.22	US$521.12
2011	US$85,675,668.33	US$75,596,071.64	US$10,079,596.70	US$223.94	US$506.60
2012	US$79,765,450.60	US$70,309,612.36	US$9,453,831.73	US$222.51	US$498.74
2013	US$73,312,840.20	US$64,552,731.09	US$8,753,021.74	US$222.32	US$492.70
2014	US$65,861,191.58	US$57,977,076.76	US$7,869,936.32	US$216.97	US$453.90
2015	US$58,633,785.16	US$51,569,645.37	US$7,054,258.12	US$212.43	US$421.73
2016	US$51,436,531.00	US$45,223,758.36	US$6,209,428.90	US$206.32	US$386.02
2017	US$45,923,843.17	US$40,467,475.84	US$5,455,540.64	US$197.40	US$346.84
2018	US$47,240,161.37	US$41,649,914.01	US$5,589,860.94	US$210.84	US$341.27
2019	US$49,905,502.21	US$43,950,172.70	US$5,954,901.25	US$216.75	US$331.33
Forecasts					
2020	US$52,570,752.42	US$46,250,340.26	US$5,954,893.44	US$216.75	US$321.38
UCL	US$61,428,804.42	US$52,218,926.38	US$9,029,276.02	US$240.28	US$374.11
LCL	US$43,712,700.42	US$40,281,754.15	US$2,880,510.85	US$193.22	US$268.66
r^2^	0.94	0.97	0.44	0.22	0.88

MHS: mean hospital stay (in days); UCL: upper confidence level; LCL: lower confidence level.

**Figure 2.**
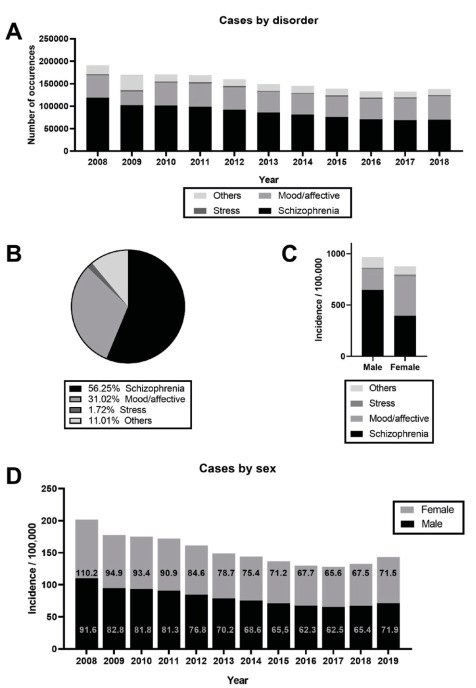
Total admissions by year of different disorders (A) and total occurrences from 2008 to 2019 by disorder type (B). Total admissions for each disorder type (C) and total annual incidence (D) by sex.

Figure 2A shows total admissions/year by specific conditions. Considering the type, there was a higher occurrence of schizophrenia (1,039,602.00 cases) in comparison to mood and affective disorders (573,270.00), stress-related disorders (31,822.00) and other mental disorders (203,422.00). As showed in Figure 2B, schizophrenia accounts for 56.25% and mood and affective disorders accounts for 31.02% of total admissions.

When comparing male to female population in specific conditions (Figure 2C), only schizophrenia incidence per 100,000 inhabitants was higher for men (M: 648.1 and F: 393.9). Moreover, incidence related to mood and affective disorders (M: 200.9 and F: 384.9), stress-related disorders (M: 12.7 and F: 18.9) and other mental disorders (105.4 and F: 80.1) were higher in females. Overall, men were hospitalized more frequently than women, as total cases were 949,798.00 for the male and 898,378.00 for female population. Annual hospital incidence by sex is showed in Figure 2D.

Regarding hospitalizations per 100,000 inhabitants (Table 3), the most frequently admitted age group were older adults (aged 40 to 49 years and 50 to 59 years) followed by younger adults (30 to 39 years and 20 to 29 years). Notably, there is a trend for increase of incidence in young individuals (aged 10 to 14: r^2=^0.77 and 15 to 19: r^2^ = 0.56), whereas other age groups showed a trend to decrease incidence (aged 20 to 29: r^2=^0.56; aged 30 to 39: r^2=^0.91; 40 to 49: r^2=^0.93; 50 to 59: r^2=^0.90 and 60+: r^2=^0.76).

**Table 3.** Incidence of schizophrenia and common mental disorder by age group.

	5-9	10-14	15-19	20-29	30-39	40-49	50-59	60+
2008	0.64	4.69	39.60	111.83	172.72	200.53	178.14	87.77
2009	0.68	4.69	36.99	100.78	154.80	175.15	148.86	67.67
2010	1.10	6.63	35.21	99.81	147.27	173.47	144.51	63.84
2011	2.47	7.94	38.43	96.05	142.83	166.23	141.34	62.91
2012	3.39	8.65	38.31	89.48	131.68	152.29	133.37	58.03
2013	3.71	8.39	36.78	83.20	118.79	137.03	122.52	54.67
2014	3.69	8.95	39.45	79.42	112.35	129.96	118.81	53.07
2015	4.07	9.88	39.75	76.81	105.11	118.03	112.83	50.98
2016	4.23	10.05	42.70	75.33	97.19	108.99	105.17	47.18
2017	2.12	10.40	45.45	75.61	94.04	105.38	103.22	46.74
2018	1.64	14.11	54.06	80.67	95.36	106.62	102.47	47.52
2019	2.11	20.67	69.57	92.83	100.84	108.85	102.31	49.45

## DISCUSSION

This study aimed to evaluate the magnitude of schizophrenia and common mental disorders in the Brazilian clinical population. We report a mean of 154,009.67 hospital admissions a year, resulting in a incidence of 77.44 admissions per 100,000 inhabitants from 2008−2018 in Brazil.

According to WHO, over 970 million people are diagnosed with one mental disorder, representing 13% of the estimated population.^15^ More specifically, in Brazil, 13.09% of male and 15.78% of female population are diagnosed with mental disorders, respectively.^15^ The data here represents hospital admissions in the SUS, so it can be expected to underestimate total diagnoses in the country, since it does not account for cases without hospitalization.

Additionally, in agreement with data from Andrade et al.,^10^ reporting that more than 30% of the population of São Paulo are diagnosed with mental disorders, the region with higher admission number was the Southeast. However, higher incidence is observed in the South followed by Midwest and Southeast. This is consistent with previous reports indicating high prevalence of mental disorders in patients and cities from Rio Grande do Sul,^12^ ^,^ ^16^ the biggest state of the south region.

The Length of hospital stay for each region by year and costs from 2008 to 2017 showed a trend towards decrease. Noteworthy, Brazil underwent a major reform of the mental health system, promoting several services and interventions aiming mental health, such as the Centros de Atenção Psicossocial (Psychosocial Care Center - CAPS) and the Return Home program.^17^ Therefore, this specific units and treatment options could explain the observed decrease in length of hospital days, which should be considered a positive result from the previous mental health policy where the number of services increased, albeit were still considered unequally distributed across the country. Reinforcing this idea is a recent study collecting data from 2008 to 2015 showing that reduced psychiatric hospitalizations rates and increased CAPS coverage were observed with inverse and statistically significant association.^18^ Contrarily, incidence, total costs, length of hospital stays and overall costs increased from 2017 to 2018, in all evaluated parameters, except for mean hospital stay cost. Noteworthy, in 2017, the Brazilian government reduced the implementation of new Community Mental Health Centers and promoted changes to the mental health policy, despite contrary recommendations from the National Human Rights Council and the National Health Council, and going against the need for better funding and structure.^19^ In summary, changes were the inclusion of psychiatric hospitals, use of electroconvulsive therapy, specific changes for hospital admission of children and adolescents, and use of abstinence to treat substance abuse disorders.^20^ Although it is too early to tell, numbers from 2018 were coincidently worst one year after the beginning of policy changes, which were latter reinforced in 2019 and according to the presented forecast could result in further increase in 2020.

Data on the selected conditions indicates higher occurrence of schizophrenia compared to mood and affective disorders, stress-related disorders and other mental disorders. This directly influences total admissions by sex, as schizophrenia has higher incidence in males, culminating in men being hospitalized more frequently for the analyzed conditions than women. Although overall data seems to be independent of sex, there are differences in symptoms and behavioral patterns across ages.^21^ Young females present higher prevalence of depression and other disorders, as well as suicidal ideation compared to young males, and throughout adulthood, depression and anxiety are more prevalent in females, while males are more prone to substance abuse, and risk behavior.^22^ Regarding specific conditions, males present early onset of schizophrenia, while women have higher risk of bipolar depression. The present data indicates higher hospital admissions due to schizophrenia in men, and more admissions due to mood and affective disorders, stress-related disorders and other mental disorders in women. Importantly, genetics, physiological, psychological and social factors are among the proposed explanations of this pattern of mental health in women, which are also more frequently exposed to stressful conditions such as domestic and sexual violence.^23^

In absolute numbers, the most frequently admitted age group were older adults (aged 40 to 49 and 50 to 59 years) followed by younger adults (30 to 39 and 20 to 29 years). Notably, there is a trend towards overall decrease, albeit young individuals (aged 10 to 14 and 15 to 19) show a trend towards increase of incidence (Table 2). This increase seems related to the high prevalence of common mental disorders among adolescents in Brazilian adolescents recently reported.^24^ The authors assessed 74,589 adolescents in 1,247 schools of 124 Brazilian cities and found 30.0% prevalence of common mental disorders. Nevertheless, it has been reported that only 19.8% of young individuals with psychiatric disorder seek mental health services in Brazil.^25^ Therefore, the data presented here indicates that this age group should be considered when implementing mental health promotions strategies by public services in the country.

The reader should observe the limitations of our analysis. We chose to focus on schizophrenia and common mental disorders. Thus, we excluded specific conditions related to drug abuse, which directly impact the numbers here presented. Also, we excluded specific neurodegenerative conditions, such as dementia, mental retardation, Alzheimer or Parkinson diseases to focus on mood and behavioral disorders. Since we calculated incidence with hospital admissions, the presented numbers could be overestimating incidence, since a patient may have had more than one hospital admission a year. In addition, considering only hospital admissions, the epidemiological trends could be explained by other factors related to schizophrenia and common mental disorders that are affected outside of the hospital, such as economic instability, public policies changes and overall access to health services. Although we can only speculate that these factors could explain the observed numbers, the results highlight a special concern to future years.

In general, the present data on schizophrenia and common mental disorders indicates positive results up to 2017, with a decrease in incidence, total costs and length of hospital stays. However, several of the evaluated parameters increased from 2017 to 2019, raising concerns for future policy changes. We consider that this should be taken into account since recently the government announced a new mental health policy, with the inclusion of psyquiatric hospitals, use of electroconvulsive therapy, specific changes for hospital admission of children and adolescents, and use of abstinence to treat substance abuse disorders. These changes had divided specialists, and we believe that the data here presented is very important to consider for future directions.
